# Establishment of reference intervals of complete blood count for twin pregnancy

**DOI:** 10.1186/s12884-021-04192-8

**Published:** 2021-10-26

**Authors:** Yifan Zeng, Lei Li, Man Mao, Xinghua Liang, Min Chen, Yong Xia, Wenyin He

**Affiliations:** 1 Guangzhou Medical University, Guangzhou, China; 2 The Third Affiliated Hospital of Guangzhou Medical University, Guangzhou, China; 3 Present address: iHuman Institute, Shanghai Tech University, Shanghai, China

**Keywords:** Twin pregnancy, Complete blood count, Reference intervals, Hematology, Preterm birth

## Abstract

**Background:**

Twin pregnancy poses a high risk, and its incidence has increased in recent years. Establishment of reference intervals of complete blood count (CBC) for women with twin pregnancies during pregnancy may aid in the prognosis of adverse outcomes.

**Methods:**

The incidence of complications and the intensity associated with adverse outcomes were analyzed in 1153 cases of twin pregnancy. A total of 253 cases in the twin pregnancy reference cohort were screened from all candidates after complications and adverse pregnancy outcomes were excluded. Complete blood count data were collected during the mid- and late-term of pregnancy and analyzed using SPSS to establish the reference intervals for peripheral blood in twin pregnancy.

**Results:**

Premature rupture of the membrane and pelvic inflammatory disease were highly positively correlated with adverse outcomes, with OR values of 3.31 and 3.81, respectively. Within the interval population with normal outcomes, red blood cell (RBC), hemoglobin (HGB), hematocrit (HCT), and platelet (PLT) values were lower in twin-pregnant women during gestation than in healthy nulligravida women, but the levels of white blood cells (WBC), neutrophils (NEU), and the NEU% increased, especially in the mid-term. The reference intervals of late-term pregnancy were validated using 20 twin pregnancies samples, and then utilized to determine the distinctive CBC characteristics in preterm birth (PTB) pregnancy. Absolute WBC and NEU values increased in PTB pregnancy based on our established reference intervals, which suggests that these may might be prognostic indicators of this adverse outcome.

**Conclusion:**

Establishing the reference interval of blood cell-related indicators of normal twin pregnancy is helpful for the monitoring and prognosis of gestation.

## Introduction

With the gradual relaxation of one-child policy in China in recent decades [1], there has been an increase in multiple pregnancies, which is primarily related to progress in the treatment of infertility and assisted reproductive technologies (ART) [2, 3]. Multiple pregnancies are associated with adverse maternal outcomes, including increased rates of preeclampsia, pregnancy-induced hypertension, maternal anemia, and venous thrombosis [4, 5]. Twin pregnancy is top 10 risk factor for stillbirth, which remained high in 2015 in China and ranked in the top five worldwide [6].

Routine blood tests are essential in the diagnosis of diseases in the clinic, such as anemia and infection. However, without correct reference intervals, there is an increased risk of missing significant changes due to pathological conditions and erroneous interpretations of regular changes as a pathological event [7, 8]. Although the physiological and biochemical changes in pregnancy influence many laboratory tests and the changes in normal laboratory values induced by pregnancy are well known [9, 10], few studies have been conducted to determine blood reference intervals for normal singleton pregnant women [11–13]. Changes in routine peripheral blood parameters insingleton pregnancy have been reported. Red cell mass increases during pregnancy but the plasma volume increases more, resulting in relative anemia. These changes lead to dilution anemia, which is associated with lower hemoglobin (HGB) levels, hematocrit (HCT) values, and red blood cell (RBC) counts [9, 10, 14]. A stable higher upper reference limit of white blood cell (WBC) count during pregnancy has also been reported. The WBC count is known to increase in pregnancy, thus limiting the use of this parameter as a marker for infection during pregnancy [10, 14]. Use of the results of healthy nonpregnant women as the reference interval may lead to the misdiagnosis of anemia or infection in a normal pregnancy, resulting in elevated medical costs and even unnecessary and potentially dangerous therapeutic actions.

Although twin pregnancy has a high risk of adverse outcomes and an increasing incidence rate, it was not included in the above-mentioned studies. Therefore, it is essential to develop reference intervals for normal twin pregnancies. As early as 1995, Blickstein described the difference in hemoglobin levels between twin and singleton pregnancies [15]. However, no comprehensive blood reference interval has been established to date for twin pregnancy without complications or adverse outcomes. Establishing accurate blood references for twin pregnancy may be extremely important for facilitating accurate clinical diagnoses, such as ablation placentae, appendicitis, premature rupture of membranes and preeclampsia, and ensuring timely access to risk-appropriate obstetric and neonatal care, which help reduce mortality and morbidity.

The correct interpretation of laboratory tests requires accurate reference intervals from an appropriate population. Based on the exclusion of twin pregnancies with complications and/or adverse outcomes, we sought to develop laboratory blood reference intervals from 120 normal Chinese women with twin pregnancies in the mid- and late-term in a single institution with virtually identical operators, equipment, and methodologies. We used these results to create reference intervals and tested them with blood samples from 20 women with twin pregnancies. After confirming the accuracy of the reference intervals, we found that the RBC, HGB, HCT, and PLT levels decreased in women pregnant with twins, while those of WBC and NEU levels increased. These results suggest that these indices may be prognostic indicators of some adverse outcomes.

## Materials and methods

### Patients

In this retrospective study, 1153 women ranging from 20 to 40 years old with twin pregnancies who were admitted to obstetric units between January 2015 and December 2018 were consecutively recruited and studied.

The complications and outcomes of twin pregnancies were investigated in these 1153 women. Complications of the pregnancies were considered based on the diagnosis from the clinic via Laboratory information system (LIS), and their incidence was analyzed as long as they were identified in one candidate. The maternal and neonatal outcomes considered in the present study were as described by Sun [16] and included preterm birth, hysterectomy, blood loss, transfusion, uterine rupture, infection, shock, sepsis, ICU transfer, length of hospital stay, ICU stay, maternal death, intrapartum stillbirth, fetal death, fetal distress, 1 min Apgar score < 5, 5 min Apgar score < 7, neonatal asphyxia and neonatal death. It was generally considered that delivery after 37 weeks of gestation and a newborn weight of 2500 g were basic criteria for a normal singleton pregnancy outcome. However, the following modifications to the delivery gestation week and newborn weight were applied in this study for twin pregnancy cases: women whose delivery time was earlier than 36 weeks of pregnancy or whose newborns weighed less than 2000 g were also included in the adverse pregnancy outcome group, and the remaining women were considered to have had a normal outcome.

The association between the identified complications and adverse pregnancy outcomes was analyzed by calculating the odds ratio (OR). Individuals who suffered complications or adverse pregnancy outcomes were excluded from the reference cohort.

### Establishment of reference intervals

The complete blood count (CBC) data of the selected reference cohort were retrieved from the Sysmex XN9000 analysis system, and analyzed using Statistical Package for Social Sciences (SPSS) version 23.0 software (IBM Corp., Armonk, NY, USA). Based on the Clinical and Laboratory Standards Institution (CLSI) guideline recommendations of the inclusion of 120 reference subjects for the establishment of reference intervals, we randomly assigned 138 cases of 158 twin-pregnant women as the reference cohort. The CBC parameters of these pregnancies at each time point were entered into an Excel database and sorted in ascending order. Reference intervals were established using nonparametric analysis [17–20]. The identification of possible outliers was performed using box plot analysis. According to CLSI/NCCLS C28-A2, outliers were excluded from each group when the D/R ratio was over 1/3, where D is the absolute difference between an extreme value and the next largest value, and R is the range of all observations.

The normal distribution assumptions for CBC data were ascertained using the Kolmogorov-Smirnov test. The descriptive statistics are presented as the means and standard deviation (SD) (x ± 1.96 s) for normally distributed data [21]. Since the reference cohort has been selected based on the exclusion criterias, normal ranges was used including 95% of the measured values and excluding the lowest and the highest 2.5%. P2.5 and P97.5 were used as the lower and upper limits of the reference interval, respectively. Thus, the reference intervals of particular indexes (RBC, HGB, HCT, PLT, WBC, NEU, NEU%, lymphocytes(LY), LY%) in late-term twin pregnancy were obtained.

These values were compared with well-established reference intervals (WS/T 405–2012) developed for the Chinese population. The reference population of this official reference intervals is residents selected from 6 regions of northeast, north, northwest, east, south and southwest China. Each region had a total of approximately 720 reference individuals, the age range was from 20 to 79 years old, and the number of reference individuals per 10-year age was at least 120. Based on questionnaire surveys, physical examinations, laboratory examinations and imaging examinations, the selected reference individuals met the following requirements: consciously healthy, no disease, no recent surgery or medication, no recent blood donation or blood loss, BMI > 18.5, no alcoholism or alcoholism, no severe blood loss, no heavy exercise or physical work, no chronic physical and chemical damage or long-term exposure to chemical substances, no excessive menstrual flow, and no pregnancy or lactation. Significant differences in values of our established reference intervals and official control were determined using a two-tailed, paired t-test analysis or one-way ANOVA in an SPSS 23.0 analysis package. Differences with *p* < 0.05 were considered significant.

### Verification of established reference intervals

Twenty clinical samples with normal gestational outcomes were randomly chosen from 158 candidates, except for the reference cohort, and processed for verification of the established reference intervals. The index of these 20 samples was compared with the established reference intervals that needed to be verified. If no more than two measured values were out of the reference range, the reference intervals were used directly. If three or more values were out of range, it was necessary to refilter the other 20 subjects in the population and reperform the above comparison to assess the result validation. If there were still three or more measured values out of range, the reference interval was reestablished [22].

### Analysis of CBC data of the PTB and PPH groups based on the established reference intervals

Based on the incidence analysis, PTB and PPH were found as the two main adverse outcomes of twin pregnancy in our center. According to the delivery time and maternal blood loss, the PTB group (live deliveries during 30—35 weeks of gestation) and PPH group (blood loss > 600 mL) were generated from the 1153 women with twin pregnancies. The late-term CBCs of these two groups were reanalyzed based on the established reference intervals to reveal some potential indicators of adverse outcome prediction.

## Results

### Clinical characteristics of twin pregnancies and definitions of exclusion criteria

A diagnosis of 21 pregnancy complications was present in 1153 cases of twin pregnancy, the incidence of which was then analyzed (Table 1). Diabetes, premature rupture of membranes, placental abruption, preeclampsia, anemia, hepatitis B carrier status, and thyroid dysfunction had higher incidence rates in twin pregnancies than singleton pregnancies, which indicated that twin pregnancy was one of the high-risk factors in pregnancy. Diabetes mellitus was the most prevalent complication(21.3%), followed by premature rupture of membranes and anemia (16.0%) and scarred uterus (10.8%).

**Table 1:** Prevalence of the complications in twin pregnancies and its association to pregnancy outcome based on univariate analysis

Complications	Amount	Percentage(%)	Outcomes			OR Value
			Adverse	Normal	Lost to follow up	
**TTTs**	**33**	**2.9**	**33**	**0**	**/**	**–**
**Hypertension**	**35**	**3**	**30**	**5**	**/**	**2.67**
**Preeclampsia**	**79**	**6.9**	**66**	**13**	**/**	**2.2**
**FGR**	**13**	**1.1**	**11**	**2**	**/**	**2.49**
**Diabetes mellitus**	**246**	**21.3**	**168**	**78**	**/**	**0.99**
**Polyhydramnios**	**13**	**1.1**	**10**	**3**	**/**	**1.51**
**Oligohydramnios**	**33**	**2.9**	**23**	**10**	**/**	**1.05**
**HBV carriers**	**62**	**5.4**	**43**	**19**	**/**	**1.03**
**PROM(premature rupture of membrane)**	**184**	**16**	**165**	**19**	**/**	**3.31**
**Scarred uterus**	**124**	**10.8**	**101**	**23**	**/**	**1.87**
**Chorioamnionitis**	**13**	**1.1**	**12**	**1**	**/**	**5.4**
**Anemia**	**185**	**16**	**111**	**68**	**6**	**0.79**
**Infection**	**24**	**2.1**	**17**	**7**	**/**	**1.1**
**Dysthyroid**	**50**	**4.3**	**37**	**13**	**/**	**1.28**
**PID(pelvic inflammatory disease)**	**28**	**2.4**	**17**	**2**	**9**	**3.81**
**Total**	**1153**					

All women with twin pregnancies underwent a planned cesarean delivery. The outcomes weregrouped into adverse and normal outcomes (the remaining outcomes) based on the criteria described in Section 2.1 of the Materials and Methods. According to single-factor analysis, TTTs, hypertension, preeclampsia, selective fetal growth restriction, premature rupture of membranes and placental abruption, scarred uterus, chorioamnionitis, and pelvic inflammation were high-risk factors for adverse outcomes in women pregnant with twins (Table 1). Chorioamnionitis showed the most significant positive correlation with adverse outcomes, with an OR value of 5.40. Premature rupture of the membrane and pelvic inflammatory disease were also highly positively correlated with adverse outcomes, with OR values of 3.31 and 3.81, respectively. However, the OR value of anemia was 0.79, which indicates that the diagnosis of anemia based on the existing reference intervals appeared to be a protective factor. This finding increases our suspicion about whether this type of diagnosis of anemia in twin pregnancy is appropriate.

**Fig. 1:**
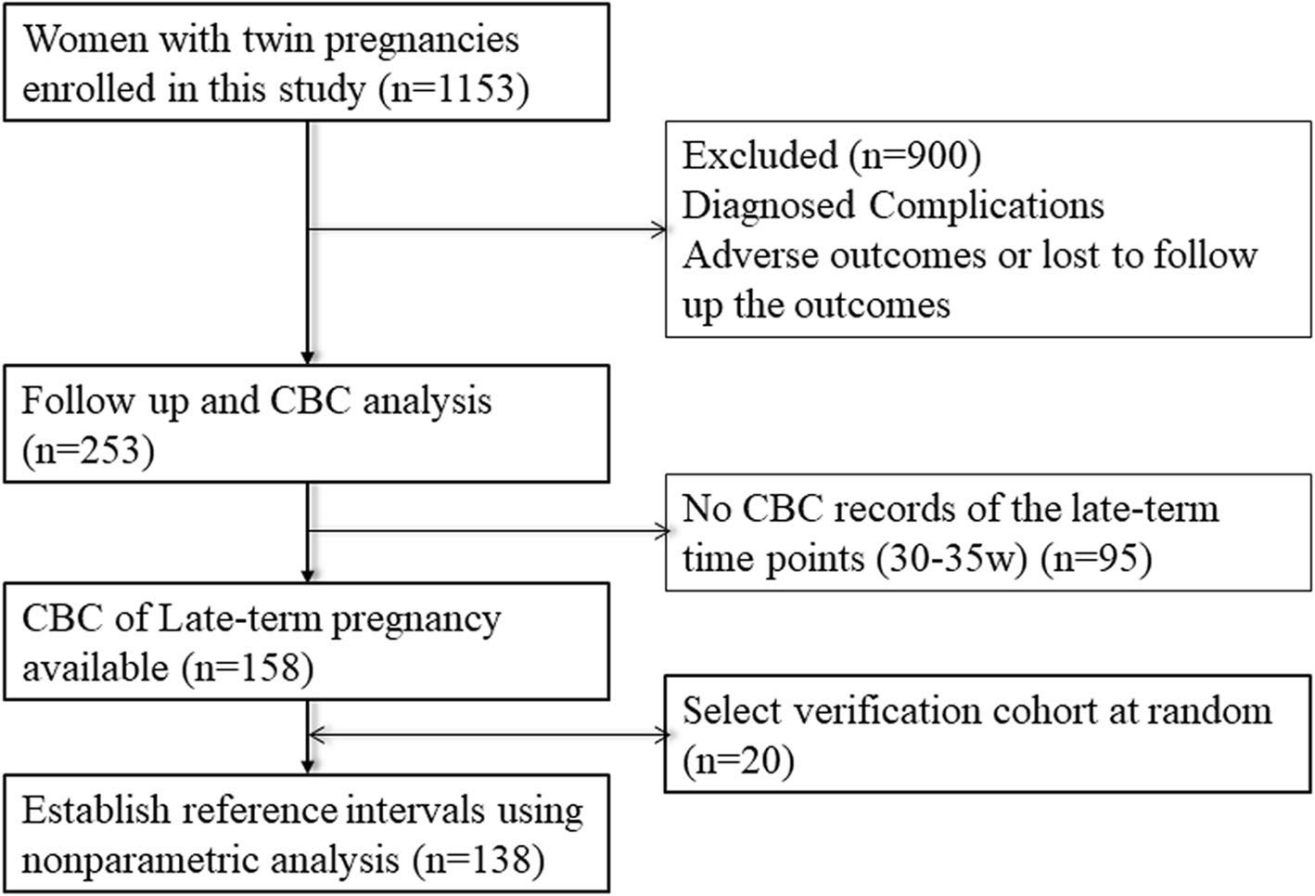
Flow diagram of reference subjects in the retrospective cohort study

After screening the clinical records of these pregnant women, 253 cases without complications or adverse outcomes were selected as the reference cohort (Fig. 1). However, when looking up the CBC records of these 253 cases in the LIS, only 158 records at 30—35 w (late-term) and 98 records at 20—25 w (mid-term) of gestation were available.

### Clinical laboratory analyses of the complete blood count of the reference cohort

The CBC data of the reference cohort during mid- and late-term pregnancy were retrieved from the Sysmex XN9000 analysis platform. Only 98 and 158 cases could be tracked, respectively. The mean pregnancy duration of this group was 36.8 weeks, and the mean dates of CBC analysis were 23 0/7 w and 32 4/7 w. The above parameters were all normally distributed based on the Kolmogorov-Smirnov test, and the mean values ± SD are shown in Table 2.

**Table 2:** Mean value comparison of nine parameters of cbc between mid-term and late-term of women with twin pregnancies

Index	Reference Intervals of Chinese Healthy Nonpregnant Female	Mean Value of Mid-term Group	Mean Value of Late-term Group	Out-of-range Percentage^a^
Number of Subject	–	86	138	
Red Blood Cells (10^12^/L)	3.80 ~ 5.10	3.58 ± 0.64	3.85 ± 0.75	60.00%
Hemoglobin (g/L)	115.00 ~ 150.00	108.70 ± 17.30	112.60 ± 24.10	65.00%
Hematocrit (%)	35.00 ~ 45.00	32.90 ± 5.10	34.40 ± 6.60	65.00%
Platelets (10^9^/L)	125.00 ~ 350.00	231.00 ± 110.00	227.00 ± 110.00	0.00%
White Blood Cells (10^9^/L)	3.50 ~ 9.50	11.36 ± 4.92	9.76 ± 5.00	50.00%
Absolute Neutrophils (10^9^/L)	1.80 ~ 6.30	8.54 ± 4.17	7.17 ± 4.17	60.00%
Percentage of Neutrophils (%)	45.00 ~ 75.00	74.90 ± 8.60	72.70 ± 11.40	55.00%
Absolute Lymphocytes (10^9^/L)	1.10 ~ 3.20	1.94 ± 0.88	1.80 ± 0.98	60.00%
Percentage of Lymphocytes (%)	20.00 ~ 50.00	17.43 ± 7.52	19.03 ± 10.52	60.00%

^a^It indicates the percentage of regarding verification values that were out of the reference range of healthy nulligravid

As the table indicates, the mid- and late-term pregnant women with twin pregnancies had lower levels of RBCs, HGB, HCT and PLT than healthy nulligravida women, and the difference was statistically significant (*P* < 0.05). RBC, HGB and HCT increased with gestation from mid- to late-term. The WBCs, NEU, and NEU% in the mid- and late-term pregnancy groups were higher than the healthy nulligravida group, and statistical significance (*P* < 0.05) was shown at the mid-term. The absolute WBC and NEU counts were 11.36 ± 4.92 and 9.76 ± 5.00 at mid-term gestation, respectively, and decreased to 8.54 ± 4.17 and 7.17 ± 4.17 at late-term gestation, respectively. During pregnancy, we found that the absolute count of LY was stable, but the LY% of the reference decreased in both the mid- and late-term compared with that of healthy nulligravida and the difference in LY% in the mid-term was statistically significant (*P* < 0.05). In conclusion, based on healthy Chinese nonpregnant females, the reference intervals of RBCs and PLT in twin pregnancy decreased, and the WBCs and NUC counts increased during pregnancy.

### The established reference intervals for twin pregnancy achieved verification

**Fig. 2:**
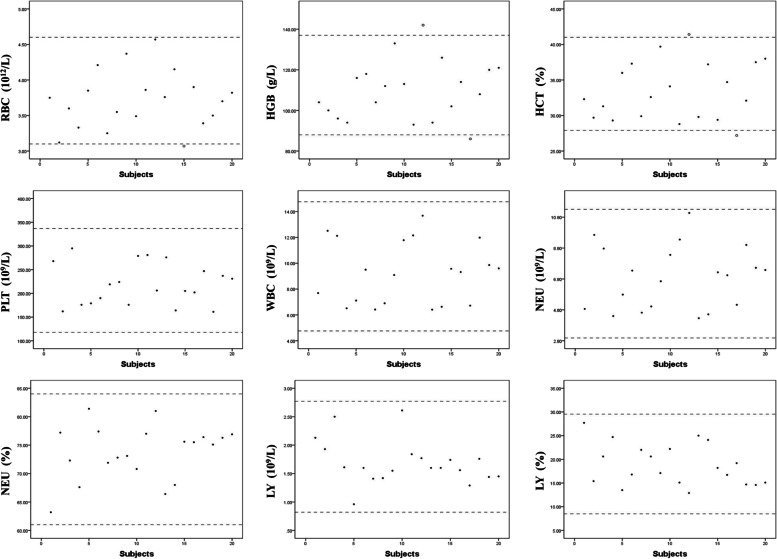
Verification of the established reference intervals. The X-axis indicates different individuals in the verification population. The black dotted line indicates the upper limit and the lower limit of the reference range for each index of twin pregnancy. The solid point represents subjects who is regarding values were within the range of reference intervals. Hollow point represent subjects who are regarding values that were out of the range of reference intervals. The slender blue dotted line indicates the upper limit and the lower limit of the reference range for each index of healthy nulligravid

The requirements of the CLSI suggest that any laboratory should have its own reference intervals if no more than two results were outside the proposed reference interval when 20 samples are uesd to evaluate a new reference interval. Therefore, a small group of 20 randomly chosen samples chosen randomly, as described in the Methods, was used to assess the reference interval for late-term pregnancy (Fig. 2). The HGB and HCT indexes of two different samples exceeded the upper and lower limits of the reference range of the twin pregnancy individually, and the RBC index of one sample was lower than the lower limit. All of the other values fell within the established reference range. Verification was achieved to apply the reference intervals directly.

However, when using the healthy Chinese female population reference intervals to analyze these 20 women in the validation cohort, 12 (60.0%) women had out-of-range RBC counts, and 13 (65.0%) women had out-of-range HGB and HCT values. All of these out-of-range values were below the lower limit of the reference range. From the perspective of WBC counts, 10 (50.0%) women in the group had out-of-range WBC counts,12 (60.0%) and 11 (55.0%) women had out-of-range NEU counts and NEU%, and 12 (60.0%) women had out-of-range LY values based on the Chinese healthy female population reference intervals (Fig. 2). These findings demonstrated the necessity of establishing reference intervals for twin pregnancy.

### Indexes of CBC might be potential indicators of adverse outcomes of twin pregnancy

**Fig. 3:**
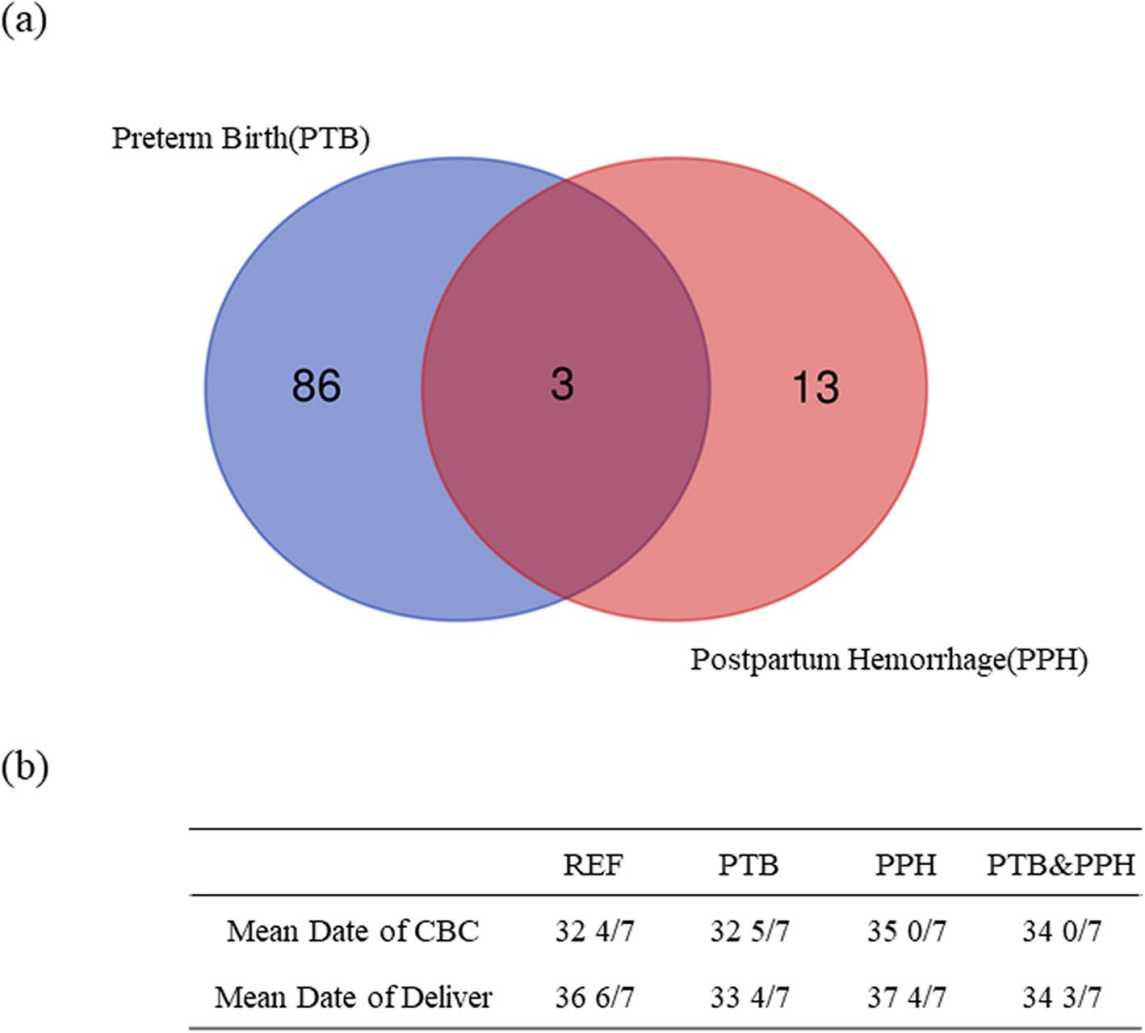
Outcomes analysis of twin pregnancy. **a** Venn diagram of the population with adverse outcomes of PTB and PPH. **b** Characteristics of the population with different gestation outcomes. REF, pregnancy with a normal outcome; PTB, pregnancy with preterm birth; PPH, pregnancy with postpartum hemorrhage

**Fig. 4:**
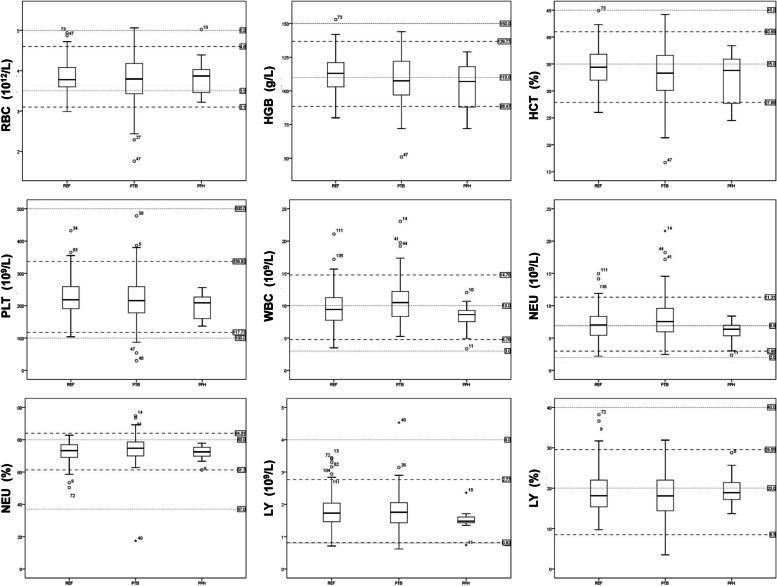
Box plot of gestational outcome-specific CBC ranges for RBC, HGB, HCT, PLT, WBC, NEU, NEU%, LY, and LY%. Each box plot contains the middle 50% of the data. The upper edge of the box indicates the 75th percentile, and the lower edge indicates the 25th percentile. The horizontal bar in the middle of each box plot represents the median value. The whiskers extending from the box plot represent the range of values obtained, excluding outliers. Circles and asterisks outside the ends of the whiskers indicate outliers (1.5× the interquartile range) and extreme values (3.0 × the interquartile range), respectively. Thebold black dotted line indicates the upper limit and the lower limit of the reference range for each index of twin pregnancy. The slender blue dotted line indicates the upper limit and the lower limit, respectively, of the reference range for each index of healthy nulligravid

After establishing the reference intervals, we further assessed their application potential in clinical diagnosis. Eighty-nine cases of twin pregnancy had a PTB during the period of 30—35 weeks of gestation, and 16 cases who suffered PPH were analyzed; 3 of these women also had a PTB (Fig. 3). We excluded the three overlapping cases to identify a single characteristic indicator relating to the particular outcome. The mean dates of the first CBC records following admission of the women to our center were 32 5/7 w and 35 0/7 w of gestation, for the PTB and PPH groups, respectively, and the mean dates of delivery were 33 4/7 w and 37 4/7 w, respectively. Based on the statistical analysis of CBC bwtween the REF, PTB, and PPH groups (Table 3), we found that the levels of HGB and HCT significantly decreased in the PTB group (*p* < 0.05), and absolute WBC and NEU increased in the PTB group. The PPH group showed a significant decrease in HGB (*p* < 0.05) but no significant differences in other indexes (Fig. 4).

**Table 3:** Multiple comparison of REF, PTB and PPH population

Dependent Variable			Mean Difference	Standard Error	Significance	95% Confidence Intervals	
						Lower Limit	Upper Limit
RBC	1^#^	2	.04704	.06138	.444	−.0738	.1679
		3	−.03217	.13217	.808	−.2925	.2281
	2	1	−.04704	.06138	.444	−.1679	.0738
		3	−.07921	.13631	.562	−.3477	.1892
	3	1	.03217	.13217	.808	−.2281	.2925
		2	.07921	.13631	.562	−.1892	.3477
HGB	1	2	5.0502*	1.9687	.011	1.173	8.927
		3	8.7210*	4.2389	.041	.373	17.069
	2	1	−5.0502*	1.9687	.011	−8.927	−1.1173
		3	3.6708	4.3717	.402	−4.939	12.280
	3	1	−8.7210*	4.2389	.041	− 17.069	−.373
		2	−3.6708	4.3717	.402	− 12.280	4.939
HCT	1	2	1.3544*	.5362	.012	.298	2.410
		3	1.6898	1.1546	.145	−.584	3.964
	2	1	−1.3544*	.5362	.012	−2.410	−.298
		3	.3353	1.1908	.778	−2.010	2.680
	3	1	−1.6898	1.1546	.145	−3.964	.584
		2	−.3353	1.1908	.778	−2.680	2.010
PLT	1	2	8.5851	8.1792	.295	−7.523	24.693
		3	28.3364	17.6114	.109	−6.347	63.019
	2	1	−8.5851	8.1792	.295	−24.693	7.523
		3	19.7513	18.1632	.278	−16.018	55.521
	3	1	−28.3364	17.6114	.109	−63.019	6.347
		2	−19.7513	18.1632	.278	−55.521	16.018
WBC	1	2	−1.12110*	.38257	.004	−1.8745	− 3677
		3	1.58468	.82374	.056	−.0376	3.2069
	2	1	1.12110*	.38257	.004	.3677	1.8745
		3	2.70578*	.84955	.002	1.0327	4.3788
	3	1	−1.58468	.82374	.056	−3.2069	.0376
		2	−2.70578*	.84955	.002	−4.3788	−1.0327
NEU	1	2	−1.06282*	.34617	.002	−1.7446	−.3811
		3	1.21539	.74537	.104	−.2525	2.6833
	2	1	1.06282*	.34617	.002	.3811	1.7446
		3	2.27821*	.76872	.003	.7643	3.7921
	3	1	−1.21539	.74537	.104	−2.6833	.2525
		2	−2.27821*	.76872	.003	−3.7921	−.7643
NEU%	1	2	−1.64563	.94321	.082	−3.5031	.2119
		3	.83935	2.03089	.680	−3.1602	4.8389
	2	1	1.64563	.94321	.082	−.2119	3.5031
		3	2.48497	2.09453	.237	−1.6399	6.6098
	3	1	−.83935	2.03089	.680	−4.8389	3.1602
		2	−2.48497	2.09453	.237	−6.6098	1.6399
LY	1	2	−.02621	.07010	.709	−.1643	.1118
		3	.26836	.15094	.077	−.0289	.5656
	2	1	.02621	.0701	.709	−.1118	.1643
		3	.29457	.15567	.060	−.0120	.6011
	3	1	−.26836	.15094	.077	−.5656	.0289
		2	−.29457	.15567	.060	−.6011	.0120
LY%	1	2	1.17497	.73010	.109	−.2629	2.6128
		3	−.50177	1.57205	.750	−3.5977	2.5941
	2	1	−1.17497	.73010	.109	−2.6128	.2629
		3	−1.67674	1.62130	.302	−4.8697	1.5162
	3	1	.50177	1.57205	.750	−2.5941	3.5977
		2	1.67674	1.62130	.302	−1.5162	4.8697

^*^indicate statistical significance (*p* < 0.05)

^#^Group 1,2,3 represent REF, PTB and PPH population respectively

**Fig. 5:**
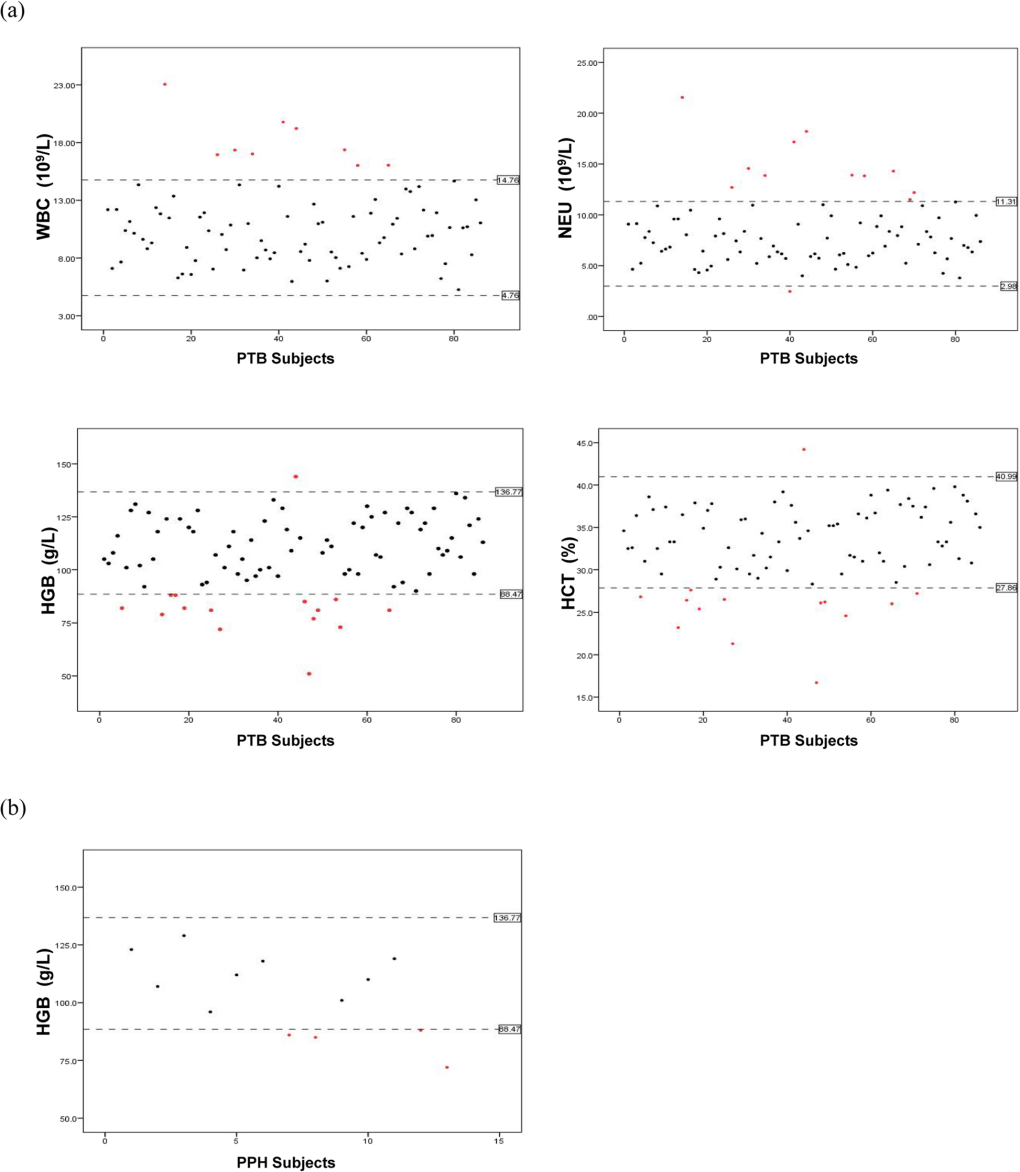
Individual distribution of responding CBC indexes with a significant difference between diverse gestation outcomes. The X-axis indicates different individuals of the PTB (**a**) or PPH (**b**) population. The black dotted line indicates the upper limit and the lower limit of the reference range for each index of twin pregnancy. The black point represents subjects who are regarding values were within the range of reference intervals. The red point represents subjects who are regarding values that were out of the range of reference intervals

Using the established reference intervals for twin-pregnant women in this study, 10.47% (9/86) of the PTB population had WBC values beyond the upper limit during late pregnancy, and 12.79% (11/86) had NEU values beyond the upper limit. Conversely, 17.44% (15/86) of the PTB population had HGB and/or HCT values that were out of range, 14 of whom had values below the lower limit and who should be considered to have any grade of anemia (Fig. 5A). Otherwise, 30.77% (4/13) of the PPH population were below the lower limit of the established HGB reference intervals (Fig. 5B).

## Discussion

Laboratory tests are often requested during pregnancy to exclude pathological complications that may affect maternal or fetal health [23, 24]. Diagnostic accuracy is based on the evaluation of results in relation to accurate reference values [25]. Although changes in normal laboratory values induced by pregnancy are well known, few studies have been conducted to establish reference intervals for pregnant women, especially those with twin pregnancies [7, 10–13]. In this study, we report x ± 1.96 s as reference intervals for CBC parameters in twin pregnancy. We found that the RBC, HGB, HCT, PLT levels and the LY% in mid- and late-term pregnant women with twin pregnancies were lower than those in healthy nulligravida women, while the WBC, NEU counts and NEU% in mid- and late-term twin pregnancy were higher [7, 10, 13]. We used these results to create reference intervals and tested our reference intervals using blood samples from 20 women with twin pregnancies. After confirming the correctness of the reference intervals, we found that the levels of RBCs, HGB, HCT, and PLTs decreased in women with twin pregnancies, and WBCs and NEUs increased, which suggests that these factors may be prognostic indicators of this adverse outcome. The establishment of suitable reference intervals for women with twin pregnancies has the potential to improve diagnostic quality, which could lead to increased survival, reducing unnecessary treatment and cost savings.

We Initially found that 16.0% of twin-pregnant women were diagnosed with anemia, the OR value of which for adverse outcomes was 0.79 (Table 1). These values strongly suggested that the use of the HBG level of healthy Chinese nulligravid individuals may result in incorrect interpretations in the clinic. The development of an accurate set of CBC reference intervals for twin pregnancies is urgently needed. Based on the reference intervals established in this study, we found that RBC, HGB, HCT, and PLT levels decreased during normal twin pregnancy during the mid- and late-term, which is consistent with findings from studies of singleton pregnancy [10, 11]. The MCV was unaffected by twin pregnancy and remained the same as the levels in healthy nulligravida women, which indicates that RBC, HGB, and HCT levels were affected by hemodilution rather than nutritional deficiencies [9]. Consistent with other studies of singleton pregnancy, we inferred that this result occurred due to the expansion of plasma volume during pregnancy with a concomitant lower expansion in red-cell volume [14]. RBC, HGB and HCT showed the lowest values in the mid-term, and these values increased slightly in the late-term. This result may be attributed to the fact that as a pregnancy develops from mid- to late-term, the body activates the metabolic system to compensate for the physiological change, and the blood dilution stabilizes [18, 26].

In contrast, HCT and RBC counts decreased with increasing gestational age in our data, but the absolute WBC count reference interval was elevated during pregnancy, which was due to the significant increase in NEU count during a normal twin pregnancy. This result may be related to a stress response, the redistribution of WBCs between the marginal and circulating pools or pain, nausea, vomiting, and anxiety in the absence of infection [19, 27]. WBC and NEU counts are expected to increase during pregnancy. Therefore, the reference intervals for these parameters should especially be considered with caution in the clinic. The use of these parameters should be limited to markers for infection during pregnancy in case proper reference intervals are lacking [20, 28]. We found that the increase in WBC count resulted primarily from an increase in NEU counts, which peaked in the mid-term and decreased in the late-term and was slightly different from the results of other studies. The absolute WBC and NEU counts decreased from 11.36 ± 4.92/8.54 ± 4.17 to 9.76 ± 5.00/7.17 ± 4.17 when entering the late-term of gestation, and the NEU% was stable. We found that the absolute count of LY was stable during twin pregnancy, and LY% was lower than that of healthy nulligravida women. Significantly lower values were detected in the mid-term (*P* < 0.05), which is consistent with the literature that absolute NEU counts peaked at the mid-term [14, 20, 28].

Investigation of potential risk markers of adverse outcomes has been performed at the molecular, biochemical, and metabolic levels [29–31]. Multiple pregnancies are associated with an increased risk of adverse maternal and perinatal outcomes, primarily due to obstetric complications, preterm birth, and birth weight discordance [32]. Cervical length, fetal fibronectin, and uterine activity monitoring, which used to be potential risk factors for interest, are not recommended because no new evidence has been identified to confirm their accuracy [33]. Using ultrasound screening, crown-rump length and placental location were measured to assess their association with adverse pregnancy outcomes [34–36]. The current study identified risk factors using more routine laboratory tests and determined that absolute NEU in CBC values may be a predictor of PTB. According to the established reference intervals for a twin pregnancy, NEU increased to above the upper limit (> 11.31 × 10^9^/L) in 12.79% of the PTB population. The association between NEU count and PTB needs further confirmation using evidence from a larger sample.

While the strength of this study is the large screened population (1153 cases) in one institute, the limitations hould not be overlooked. First, the sample size for reference interval establishment was small due to the high incidence of adverse outcomes in twin pregnancy, although it met the requirement of CLSI guidelines. Second, CBC data of candidates were obtained primarily from examinations in the second and third trimesters due to the retrospective nature of this study. Therefore, we may have missed the earlier changes in maternal hematology that corresponded to twin pregnancies. However, compared with the use of standard whole blood reference intervals from nonpregnant women, which may result in an increase in out-of-range values among twin pregnant women, the limitations of the current study may not have significantly affected the accuracy of the reference intervals for this population.

Twin pregnancy clinics were established in our center to provide better medical services for such a high-risk population. Currently, the reference interval we established is in a further stage of clinical verification and cannot be presented as a formal reference value inroutine blood reports, but rather as a comment. However, according to feedback from obstetricians of the twin pregnancy clinics, the reference interval allows them to better assess the status of the twin pregnant women, which enables them to eliminate an “infection” deduced by an increasing WBC count and arrange an amniocentesis, as well as to eliminate “anemia” deduced on the basis of a decreasing HGB value and avoid excessive iron supplementation. Compared with the current officially adopted reference interval, the interval we established, particularly for women with twin pregnancies, can aid obstetricians in precise clinical decision-making and reduce artificial interference. After long-term and large-scale clinical sample verification, we will upgrade the LIS to identify the application department of test and diagnosis keywords (twin, gestational age), then retrieve the reference interval of the corresponding stage and present it in the report automatically.

In summary, CBC indexes varied to different degrees with the development of twin pregnancy. The present study established reference intervals for several hematological variables in healthy women with twin pregnancies that may be used for clinical management in our center. These values should be considered for the development of region-specific reference intervals for women with twin pregnancies in China. Further validation and practice in the clinic of these established reference intervals are warranted. We hope that the presentation of these reference values will assist clinicians in distinguishing between physiological changes and pathological states during twin pregnancy, and help clinicians make effective judgments and administer treatment in a timely manner.

## Data Availability

The dataset generated and/or analyzed during this study are available from the corresponding author on reasonable request.

## Abbreviations

CBC: Complete Blood Count
RBC: Red Blood Cell
WBC: White Blood Cell
HGB: Hemoglobin
HCT: Hematocrit
NEU: Neutrophils
PLT: Platelets
PTB: Preterm Birth
PPH: Postpartum Hemorrhage
ART: Assisted Reproductive Technologies
TTTS: Twin-Twin Transfusion Syndrome
PROM: Premature Rupture Of Membrane
PID: Pelvic Inflammatory Disease
